# Structural Characterization and In Vivo Gastric Mucosal Protective Activity of a Polysaccharide from Laoxianghuang (Fermented Finger Citron) in Mice

**DOI:** 10.3390/ijms27177919

**Published:** 2026-09-05

**Authors:** Heming Liu, Cheng Zhong, Dan Yang, Yuxiao Wu, Junyun Luo, Aimei Zhou

**Affiliations:** Guangdong Provincial Key Laboratory of Nutraceuticals and Functional Foods, College of Food Science, South China Agricultural University, Guangzhou 510642, China; 851505238@stu.scau.edu.cn (H.L.); z17820688131@163.com (C.Z.); 20202145044@stu.scau.edu.cn (D.Y.); 20241145017@stu.scau.edu.cn (Y.W.); 20232145023@stu.scau.edu.cn (J.L.)

**Keywords:** Laoxianghuang polysaccharide, structural characterization, gastric mucosal protection

## Abstract

PFCP-2-1 (polysaccharide-2-1 of Finger Citron-pickled products), a water-soluble polysaccharide enriched in galacturonic acid, was isolated from Laoxianghuang by hot water extraction and purified using DEAE-52 and agarose CL-6B chromatography. Structural analysis showed that PFCP-2-1 had a smooth surface, flakes of varying sizes and shapes, irregular ellipsoidal structures, and cylindrical-like forms, which likely represent aggregated assemblies formed by the association and curling of polysaccharide chains. It was determined to have an average molecular weight of 749.38 kDa and to consist of rhamnose, galactose, glucose, and galacturonic acid (molar ratio 0.147:0.238:0.059:0.556). FT-IR spectroscopy revealed both α- and β-pyranose configurations, while methylation and NMR analyses identified 16 types of glycosidic linkages, of which 9 were the main types, consistent with a pectic-type structure. In an ethanol-induced acute gastric ulcer mouse model, PFCP-2-1 exhibited significant mucosal protection by elevating prostaglandin E2 (PGE2), transforming growth factor-α (TGF-α), and mucin 5AC (MUC5AC) levels and upregulating Occludin and zonula occludens-1 (ZO-1) expression. It also modulated the gut microbiota by increasing the abundance of *Bacteroides* and *Prevotella*_UCG-001, and enhanced short-chain fatty acid (acetate and butyrate) levels. These findings suggest that PFCP-2-1 may exhibit a protective effect against ethanol-induced gastric damage under the conditions of this experiment, suggesting its potential for further development as a functional food ingredient.

## 1. Introduction

Gastric ulcer is a common digestive disease, and epidemiological surveys indicate that its incidence has been rising year by year, seriously affecting quality of life. Ethanol is a major aggressive factor for gastric mucosal injury: excessive alcohol consumption elevates oxygen free radicals, induces oxidative damage, and ultimately leads to ulcer formation [1]. Although several drugs are clinically used to treat gastric ulcers, they often cause side effects such as gastrointestinal disturbances, hepatorenal toxicity, hypergastrinemia, and neuro-ocular damage [2]. To avoid these adverse reactions, natural bioactive compounds with gastroprotective properties have drawn increasing attention. In particular, numerous polysaccharides—such as Hericium erinaceus polysaccharide, wax gourd peel polysaccharide, and Lycium barbarum polysaccharide—have been reported to protect the gastric mucosa [3].

Laoxianghuang (also known as old citron) is a traditional fermented product unique to the Lingnan region of China. It is made from fresh Finger Citron (*Citrus medica* L. var. *sarcodactylis* Swingle) through salting, drying, cooking, sugar-soaking, herb addition, re-drying, and long-term sealed fermentation, which yields a glossy, soft texture and functions such as regulating intestinal function and relieving cough [4]. As a fermented medicinal-food resource, its polysaccharides are generated in the presence of microbial metabolism and enzymatic modification, which may confer distinct structural features and bioactivities compared with polysaccharides from non-fermented plants. However, existing studies on Laoxianghuang have mainly focused on flavor evolution, quality and safety, and manufacturing techniques [5,6,7], whereas its bioactive constituents and functional activities remain insufficiently characterized.

Our group previously found that crude polysaccharides from Laoxianghuang exhibited fermentation-time-dependent wound-healing activity on human gastric epithelial GES-1 cells, reaching a plateau after five years of fermentation. To quantify this enhancement, we compared the aqueous extracts of Finger Citron-pickled products (FCPP) at different fermentation stages in the same in vitro scratch-wound-healing assay. The 24 h repair rates for the raw material (0-year fermentation), 3-year-fermented product, and 5-year-fermented product were 63.03%, 72.52%, and 79.80%, respectively, representing relative increases of approximately 15.1% and 26.6% compared to the raw material extract (*p* < 0.05) [8]. During in vivo study, the crude FCPP aqueous extract at 100 mg/kg significantly reduced the gastric ulcer index in ethanol-induced acute gastric ulcer rats. The gastric ulcer index was 27.91%, and the ulcer inhibition rate was 42.7%, significantly lower than that of the model group (*p* < 0.01). Further purification of the 5-year-fermented product via DEAE-52 and agarose CL-6B chromatography yielded six polysaccharide fractions (PFCP, PFCP-1, PFCP-2, PFCP-3, PFCP-2-1, and PFCP-2-2). All fractions were evaluated for gastroprotective activity using an ethanol-damaged GES-1 cell model and a scratch wound-healing assay. While all purified fractions enhanced cell viability and antioxidant capacity to varying degrees, PFCP-2-1 (polysaccharide-2-1 of Finger Citron-pickled products) consistently exhibited the most potent activity. At 50 μg/mL, it achieved the best improvement in SOD activity and MDA reduction, and, at 5 μg/mL, it showed the greatest enhancement of CAT activity, both significantly outperforming the model group (*p* < 0.05). In the scratch assay, PFCP-2-1 at 50 μg/mL attained a wound-healing rate of 64.01% ± 6.85% at 24 h, which was significantly higher than the control group and comparable to the teprenone group [9]. Based on these in vitro results, PFCP-2-1 was identified as the fraction with the strongest gastric mucosal protective activity among all tested fractions, and was therefore selected for further in vivo evaluation and comprehensive structural characterization in the present study.

Currently, the gastroprotective mechanisms of natural polysaccharides are mainly interpreted from the perspectives of antioxidant, anti-inflammatory, and anti-apoptotic activities [3,10,11]. Systematic investigations into structurally defined polysaccharides, particularly regarding their roles in maintaining gastric epithelial barrier function, are still limited. Moreover, while emerging evidence suggests that polysaccharides may exert protection through the modulation of the gut microbiota and its metabolites, the involvement of gut microbiota and short-chain fatty acids (SCFAs) in polysaccharide-mediated gastric mucosal protection has not been systematically clarified.

In the present study, we first characterized the structure of PFCP-2-1 using scanning electron microscopy (SEM), atomic force microscopy (AFM), gel permeation chromatography (GPC), ion chromatography (IC), Fourier transform infrared spectroscopy (FT-IR), gas chromatography–mass spectrometry (GC-MS), and nuclear magnetic resonance (NMR). We then established an ethanol-induced gastric mucosal injury model in KM mice to measure serum SOD activity, gastric tissue levels of MDA, prostaglandin E2 (PGE2), transforming growth factor-α (TGF-α), and mucin 5AC (MUC5AC), as well as the expression of the tight junction proteins occludin and zonula occludens-1 (ZO-1). Finally, by analyzing the gut microbiota composition and SCFA profiles, we explored whether the gastroprotective effect of PFCP-2-1 is associated with the modulation of intestinal flora and SCFAs.

## 2. Results

### 2.1. Characterization of PFCP-2-1

#### 2.1.1. Morphological Structure

**Figure 1:**
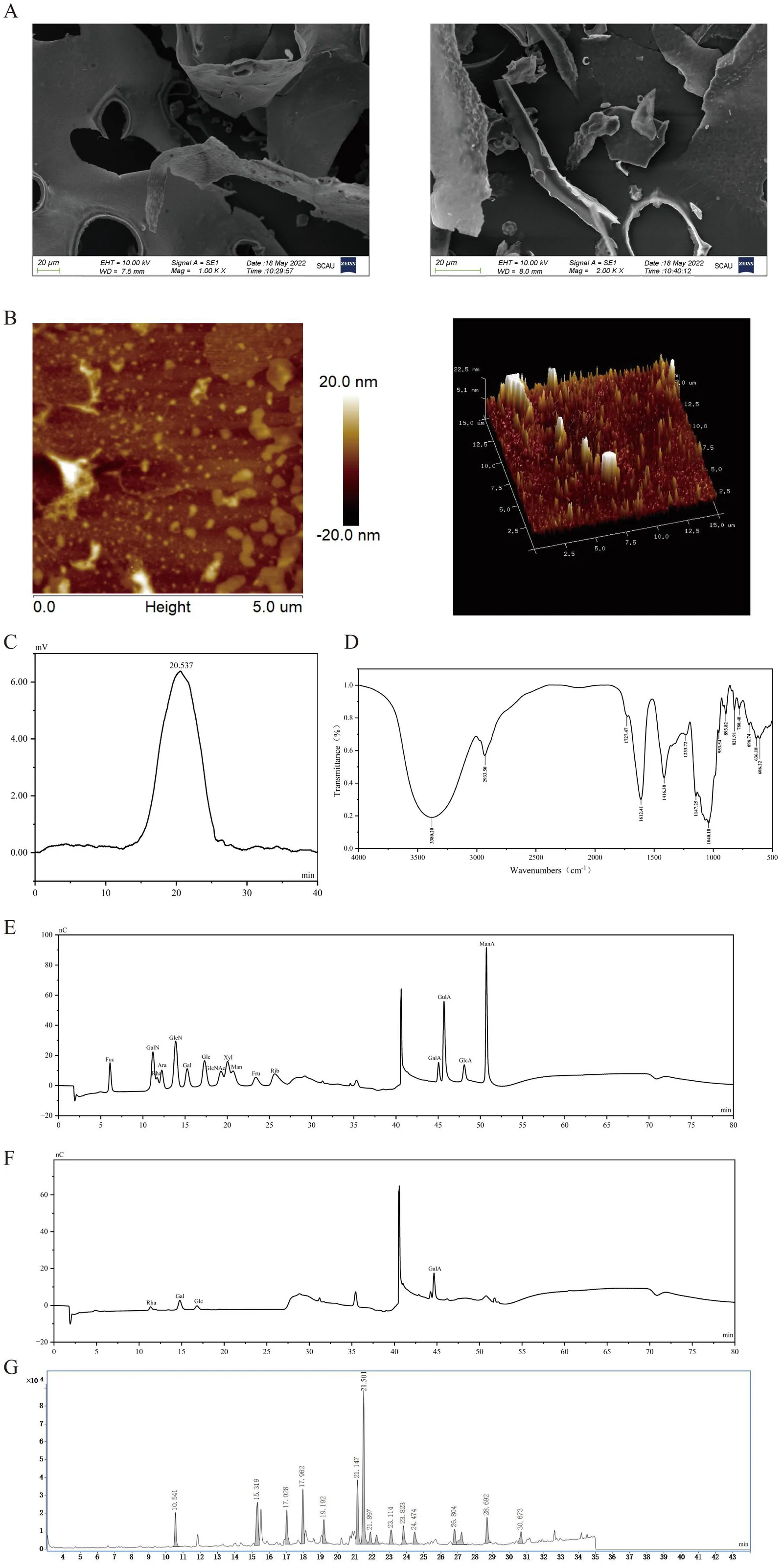
(**A**) Scanning electron micrographs of PFCP-2-1, 1000× and 2000×; (**B**) AFM diagram of PFCP-2-1; (**C**) HPSEC spectrum of PFCP-2-1; (**D**) FT-IR spectra of PFCP-2-1; (**E**) ion chromatogram of monosaccharide standard; (**F**) ion chromatogram of PFCP-2-1; (**G**) total ion chromatogram of the methylated products of PFCP-2-1.

Scanning electron microscopy was used at 1000× and 2000× magnifications to examine the microstructure of PFCP-2-1, and Figure 1A reveals that its surface was smooth and connected in a block-like manner. The morphology of PFCP-2-1 was further analyzed by AFM in tapping mode, providing clear structural images (Figure 1B). PFCP-2-1 showed sheet-like fragments of varying sizes, irregular elliptical structures, and cylinder-like forms. Individual polysaccharide chains typically have diameters of 0.1–1 nm. However, the observed structures were much larger, suggesting that PFCP-2-1 existed as aggregated assemblies formed by the association and coiling of multiple polysaccharide chains.

#### 2.1.2. Molecular Weight Analysis

As shown in Figure 1C, PFCP-2-1 exhibited a single symmetrical peak with a retention time of 20.537 min on high-performance size-exclusion chromatography (HPSEC). Using pullulan standards for calibration, its average molecular weight was estimated to be 749.38 kDa.

#### 2.1.3. Monosaccharide Composition

As presented in Figure 1E,F, PFCP-2-1 was primarily composed of rhamnose (Rha), galactose (Gal), glucose (Glc), and galacturonic acid (GalA) in a molar ratio of 0.147: 0.238: 0.059: 0.556. Galacturonic acid was the most abundant, followed by galactose, indicating that PFCP-2-1 is an acidic heteropolysaccharide.

#### 2.1.4. FT-IR Spectrum Characterization

According to Figure 1D, the infrared absorption peak observed at 3380.2 cm^−1^ was attributed to the O-H stretching vibration of hydroxyl groups, serving as a distinctive absorption marker for polysaccharides. At 2933.5 cm^−1^, the absorption peak was due to the asymmetric C-H stretching and bending vibrations of CH, CH_2_, and CH_3_ groups, whereas the absorption at 1416.38 cm^−1^ corresponded to the angular deformation of C-H bonds. The pronounced absorption bands at 1612.41 cm^−1^ and 1233.72 cm^−1^, corresponding to the stretching motions of C = O and C-O bonds, suggested that PFCP-2-1 contains uronic acids. The lack of an absorption peak near 1541 cm^−1^ demonstrated the absence of protein. Three distinct bands observed in the 1200–1000 cm^−1^ region indicated the existence of C-O-H and C-O-C bonds, implying that PFCP-2-1 contains a pyranose ring structure. The peaks at 1147 cm^−1^ and 821 cm^−1^ were indicative of the α-anomeric configuration. The peak at 821 cm^−1^ suggested the existence of α-D-Galactose, while the absorption at 893 cm^−1^ confirmed the presence of a β-D-Glucose configuration.

#### 2.1.5. Backbone Structure and Glycosidic Linkage

Methylation was used to analyze the glycosidic bond types and connectivity of the PFCP-2-1 components; following hydrolysis, reduction and acetylation were performed, and the resulting products were analyzed by GC-MS. By combining the monosaccharide composition with their ratios, the linkage patterns of the monosaccharides were established. Based on the PFCP-2-1 methylation ion flow diagram (Figure 1G), along with the mass spectral fragment information and monosaccharide composition ratios, 16 different types of glycosidic linkages were identified. According to Table 1, the 16 glycosidic linkages of PFCP-2-1 were determined to be 2,3,4-Me_3_-Rhap, 2,4-Me_2_-Rhap, 2,3,4,6-Me_4_-Glcp, 2,3,4,6-Me_4_-Galp, 3-Me_1_-Rhap, 2,4,6-Me_3_-Glcp, 2,3,6-Me_3_-Galp, 2,3,6-Me_3_-Glcp, 2,4,6-Me_3_-Galp, 2,3,4-Me_3_-Glcp, 2,3,4-Me_3_-Galp, 2,6-Me_2_-Glcp, 2,3-Me_2_-Glcp, 2,4-Me_2_-Glcp, 2,3-Me_2_-Galp, and 2,4-Me_2_-Galp, with molar ratios of 0.064, 0.108, 0.064, 0.094, 0.042, 0.105, 0.255, 0.025, 0.021, 0.030, 0.035, 0.026, 0.028, 0.033, 0.046, and 0.024, respectively. From the results, it was observed that galactose residues predominated, in agreement with the monosaccharide composition data, and no signals related to uronic acids were detected in PFCP-2-1.

#### 2.1.6. NMR Spectrum Characterization

The structure of PFCP-2-1 was analyzed using one-dimensional (^1^H, ^13^C) and two-dimensional (HSQC, ^1^H−^1^H-COSY, and NOESY) nuclear magnetic resonance spectroscopy. The main chemical shifts in PFCP-2-1 were established based on the monosaccharide composition, linkage sites, and chemical signals, as presented in Table 2.

**Figure 2:**
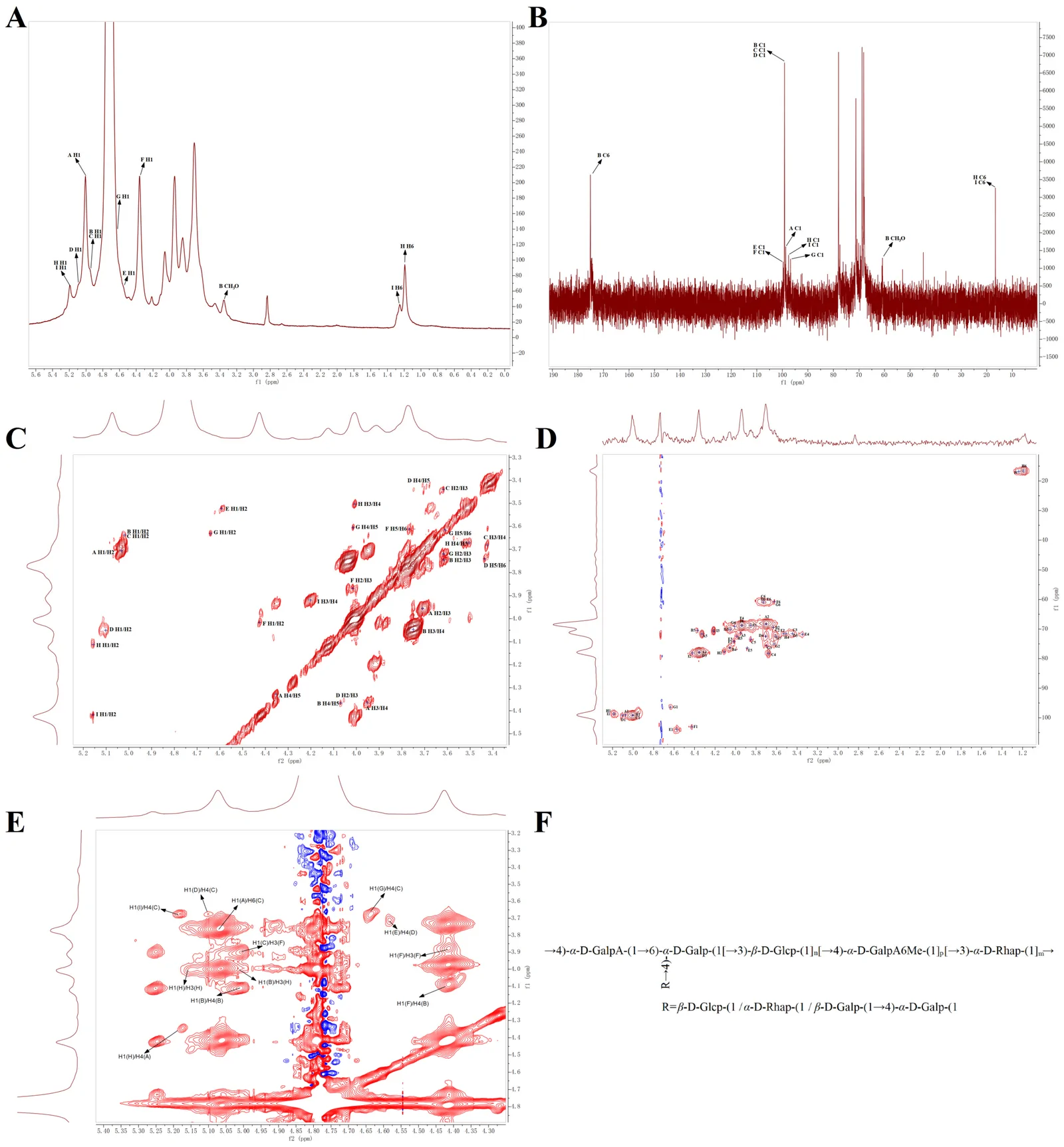
NMR spectra of PFCP-2-1 and possible chemical structure of PFCP-2-1. (**A**) ^1^H; (**B**) ^13^C; (**C**) COSY; (**D**) HSQC; (**E**) NOESY; (**F**) possible chemical structure of PFCP-2-1.

The ^1^H and ^13^C spectra of PFCP-2-1 were analyzed. As illustrated in Figure 2A, the ^1^H NMR spectrum of PFCP-2-1 revealed that the anomeric proton chemical shifts in the polysaccharide typically ranged from 4.4 to 5.2 ppm. Residues exhibiting chemical shifts exceeding 4.8 ppm were identified as possessing α-glycosidic linkages, whereas those with shifts below 4.8 ppm were characterized by β-glycosidic linkages. The spectrum displayed chemical shift signals at δ 5.07, 5.02, 5.02, 5.10, 4.58, 4.43, 4.63, 5.18, and 5.18 ppm, which were attributed to the H-1 signals of the terminal protons of residues A, B, C, D, E, F, G, H, and I, respectively. As depicted in Figure 2B, the ^13^C spectrum of PFCP-2-1 exhibited major anomeric carbon chemical shifts at δ 99.08, 99.37, 99.61, 99.37, 103.92, 103.05, 96.32, 98.70, and 98.70 ppm, which were assigned to the signals of residues A, B, C, D, E, F, G, H, and I, respectively. The signal at δ 175.03 ppm, corresponding to a uronic acid, was assigned as the C-6 signal of residues A and B. The signals observed at δ 16.63 and δ 16.84 ppm were attributed to the -CH_3_ signals of rhamnose, corresponding to the C-6 signals of residues H and I, respectively.

The other signals in the ^1^H and ^13^C spectra were analyzed by means of ^1^H−^1^H-COSY (Figure 2C) and HSQC spectrum (Figure 2D). Given the abundance of polysaccharide residues in PFCP-2-1, the paper examined the signal distribution in the ^1^H and ^13^C spectra using residue A (→4-α-D-GalpA-1→) as a representative example. The ^1^H-^1^H-COSY spectrum provided several cross-peaks at δ 5.07/3.70 ppm, δ 3.70/3.95 ppm, δ 3.95/4.36 ppm, and δ 4.36/4.33 ppm. Among these, the signal at δ 5.07 ppm was designated to residue A’s H-1, and the signals at δ 3.70, 3.95, 4.36, and 4.33 ppm were assigned to its H-2, H-3, H-4, and H-5, respectively. Based on the distribution of proton signals for residue A observed in the ^1^H−^1^H-COSY spectrum, the corresponding carbon signals for residue A were identified in the HSQC spectrum. The cross-peaks at δ 5.07/99.08, δ 3.70/68.31, δ 3.95/71.35, δ 4.36/77.86, and δ 4.33/71.35 ppm corresponded to the (H-1)/(C-1), (H-2)/(C-2), (H-3)/(C-3), (H-4)/(C-4), and (H-5)/(C-5) of residue A, respectively. Furthermore, the proton and carbon signals of the other sugar residues were assigned through a combined analysis of the ^1^H−^1^H-COSY and HSQC spectra.

The NOESY spectrum (Figure 2E) was used to establish the glycosidic linkage sequence. Inter-residue cross-peaks were observed between the H-1 of residue A and the H-6 of residue C, the H-1 of residue C and the H-3 of residue F, the H-1 of residue F and the H-4 of residue B, the H-1 of residue B and the H-3 of residue H, and the H-1 of residue H and the H-4 of residue A. These correlations defined the main chain repeating unit as →4)-α-D-GalpA-(1→6,4)-α-D-Galp-(1→3)-β-D-Glcp-(1→4)-α-D-GalpA6Me-(1→3)-α-D-Rhap-(1→, in which residues A, C, F, B, and H are arranged in a linear hetero-sequence.

In addition, intra-residue or homo-residue NOE cross-peaks were detected: H-1 of residue F correlated with its own H-3, H-1 of residue B with its own H-4, and H-1 of residue H with its own H-3. These signals indicate that residues F, B, and H each can form short homopolymeric stretches through (1→3) or (1→4) linkages. Thus, the overall main chain is a block-wise arrangement of the A-C-F-B-H hetero-unit and homo-oligomeric segments of F, B, and H. Accordingly, the backbone of PFCP-2-1 was characterized as: →4)-α-D-GalpA-(1→6,4)-α-D-Galp-(1→[→3)-β-D-Glcp-(1]_n_[→4)-α-D-GalpA6Me-(1]_p_[→3)-α-D-Rhap-(1]_m_→, where n, p, and m denote the number of repeating units in the homo-blocks of residues F, B, and H, respectively. The main chain comprises (1→6), (1→3), and (1→4) glycosidic linkages.

Following the determination of the main chain structure, the NOESY spectra of the remaining sugar residues were further analyzed. Figure 2E shows intersecting signals between E(H-1) and D(H-4), indicating that the linkage among residues E and D could be β-D-Galp-(1→4)-α-D-Galp-(1→. Cross-peaks were also detected between G(H-1) and C(H-4), between I(H-1) and C(H-4), and between D(H-1) and C(H-4), suggesting possible branch-forming (1→4) linkages at residue C, namely β-D-Glcp-(1→4,6)-α-D-Galp-(1→, α-D-Rhap-(1→4,6)-α-D-Galp-(1→, and β-D-Galp-(1→4)-α-D-Galp-(1→4,6)-α-D-Galp-(1→. Collectively, the analyses led to the proposed chemical structure of PFCP-2-1, as depicted in Figure 2F. It should be acknowledged that the relatively high molecular weight and limited solubility of PFCP-2-1 may lead to reduced signal-to-noise ratios in certain regions of the 2D NMR spectra, which is a common limitation in the structural analysis of natural polysaccharides. Nevertheless, the key correlations supporting the proposed backbone and linkage patterns were clearly identifiable and were corroborated by methylation analysis and monosaccharide composition.

### 2.2. Gastroprotective Activity of PFCP-2-1

#### 2.2.1. Effects on Body Weight, Organ Index, and Gastric Lesions

**Figure 3:**
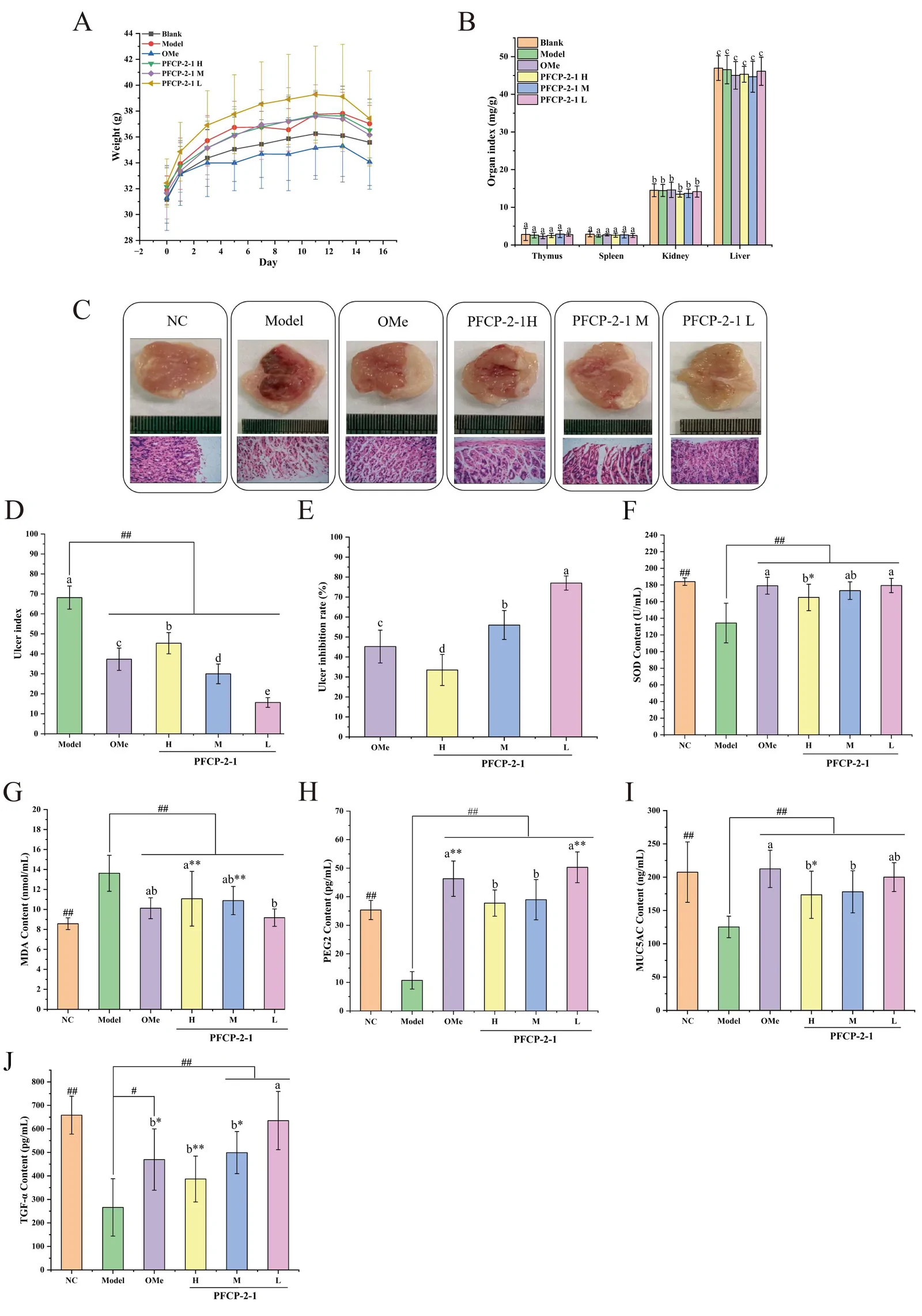
(**A**) Body weight of mice; (**B**) organ index; (**C**) photographs of mouse stomachs and H&E-stained sections (400×) of mouse gastric tissues; (**D**) gastric ulcer index; (**E**) gastric ulcer inhibition rate; (**F**) serum SOD enzyme activity; (**G**) serum MDA content; (**H**) gastric homogenate PGE2 content; (**I**) gastric homogenate MUC5AC content; (**J**) gastric homogenate TGF-α content. Different letters indicate significant differences, *p* < 0.05; Versus NC, * *p* < 0.05, ** *p* < 0.01; Versus Model, # *p* < 0.05, ## *p* < 0.01. NC: normal control group; Model: model group; OMe: omeprazole group; PFCP-2-1H: high-dose group of PFCP-2-1; PFCP-2-1M: medium-dose group of PFCP-2-1; PFCP-2-1L: low-dose group of PFCP-2-1.

KM mice were given corresponding drugs by gavage for 15 days, and the weight changes during gavage are depicted in Figure 3A. During gavage, the weight of mice in each group showed a steady growth trend. However, the body weight of mice showed a downward trend on the 15th day, which might be caused by fasting the day before dissection. On the day of dissection, the organ index of mice is shown in Figure 3B. The spleen, kidney, liver, and thymus indices showed no significant differences (*p* > 0.05) among the NC group, model group, OMe group, and the various doses of PFCP-2-1.

The gastric ulcer severity in KM mice, pretreated with PFCP-2-1 and induced by alcohol, was observed, and their ulcer index was calculated. As can be seen from Figure 3C, the NC group mice’s stomach tissues had normal macro-morphology, a smooth surface, complete mucosa, no congestion or edema, and a healthy reddish state. In the model group, the surface of gastric tissue was crimson, with obvious punctate, linear and large area strip bleeding and erosive lesions, and the ulcer injury was serious, with the ulcer index as high as 68.17 points, which was significantly higher than that of the NC group (*p* < 0.05), the results indicated that the acute gastric ulcer mouse model was successfully established. In the OMe group and PFCP-2-1 group, the surface of the gastric mucosa was slightly damaged, and a small amount of punctate or linear bleeding could be seen with naked eyes. With slight mucosal edema and congestion, the surface of the gastric mucosa was basically reddish, and the ulcer index (Figure 3D) of mice was significantly lower than that of the model group (*p* < 0.01). Among them, the ulcer index of the PFCP-2-1M and PFCP-2-1H groups were 30 points and 45.33 points respectively, which were significantly lower than those of the model group, and the ulcer inhibition rate (Figure 3E) of the two groups were 55.99% and 33.50%, respectively. However, the PFCP-2-1L group had the best preventive effect on acute ethanol-induced gastric mucosal injury, and the gastric ulcer index was only 15.67, which was significantly lower than that of PFCP-2-1M, the PFCP-2-1H group and model group (*p* < 0.01), and the ulcer inhibition rate was 77.02%, which was better than that of the OMe group (*p* < 0.01).

The results of H&E staining of mouse stomach tissues are presented in Figure 3C. In the NC group, the gastric tissue structure was clear and complete, with distinct layers, and the gastric glands were tubular, closely arranged and orderly, and no red blood cell extravasation was found. After being stimulated by ethanol, the mice in the model group suffered from serious mucosal epithelial gland shedding and damage. The structure of mucous membrane, submucosa and muscle layer was disordered, and the organizational structure was messy and loose; there was local inflammatory cell infiltration, accompanied by scattered bleeding points. Compared with the model group, the damage of gastric mucosa could be obviously improved by PFCP-2-1. Among them, the gastric glands in the PFCP-2-1L group were compact, arranged neatly and clearly, and no symptoms such as mucosal defect, inflammatory cell infiltration, redness and swelling were found, and the gastric tissue structure was the closest to that of normal mice.

#### 2.2.2. Effects on Serum Antioxidant Indicators

The results of SOD and MDA contents in mouse serum are demonstrated in Figure 3F,G. As can be seen from Figure 3F, the lowest SOD activity value in the model group was 134.32 U/mL, and the highest SOD activity value in the NC group was 183.98 U/mL, with significant difference (*p* < 0.01). After intervention with PFCP-2-1 and omeprazole, the SOD enzyme activity in the serum of mice significantly increased compared to the model group (*p* < 0.01). Meanwhile, except for the PFCP-2-1H group, which had significantly lower SOD enzyme activity compared to the NC group (*p* < 0.05), the other three groups (OMe, PFCP-2-1L, and PFCP-2-1M) showed no significant differences from the NC group (*p* > 0.05). The more effective groups were the OMe and PFCP-2-1L groups, with SOD activity values of 179.09 U/mL and 179.41 U/mL, respectively, both of which had essentially the same SOD enzyme activity (*p* > 0.05). This was followed by the PFCP-2-1M and PFCP-2-1H groups with SOD enzyme activities of 173.24 U/mL and 165.05 U/mL, respectively.

It could be clearly seen from Figure 3G that the lowest MDA content was in the NC group, which was 8.57 nmol/mL, while the highest MDA content was in the model group, which reached 13.63 nmol/mL. However, both PFCP-2-1 and omeprazole interventions effectively reduced the serum MDA levels in mice with ethanol-induced acute gastric injury (*p* < 0.01). Among the groups, the serum MDA level in the PFCP-2-1L group was the lowest (9.18 nmol/mL), followed by the OMe group (10.12 nmol/mL), with no significant difference between the two groups (*p* > 0.05), and there was also no significant difference compared with the NC group (*p* > 0.05). It showed that the low dose of polysaccharide from Laoxianghuang can reduce the MDA value in the serum of mice.

#### 2.2.3. Effects on Gastric Mucosal Defense Factors

As depicted in Figure 3H, the NC group was 35.75 pg/mL, while that in the model group was 10.77 pg/mL, which was significantly reduced (*p* < 0.01). Compared with the content of PGE2 in the model group, the content of PGE2 in the gastric homogenate of mice increased significantly after the intervention of PFCP-2-1 and omeprazole (*p* < 0.01). Among them, the content of PGE2 in the stomach tissue of the OMe group was 45.39 pg/mL, and that of the PFCP-2-1H, PFCP-2-1M and PFCP-2-1L groups were 39.93 pg/mL, 42.36 pg/mL and 51.25 pg/mL in turn. The PGE2 levels in all groups were significantly higher than those in the model group (*p* < 0.01), while only the PFCP-2-1L group and the OMe group had PGE2 levels significantly higher than the NC group (*p* < 0.01). Importantly, the PFCP-2-1L group had the best effect of increasing PGE2 content, which was 375.85% higher than that in the model group (*p* < 0.01).

As shown in Figure 3I, the MUC5AC content in the model group was 125.29 ng/mL, significantly lower than that in the NC group (207.56 ng/mL) (*p* < 0.01). After intervention with PFCP-2-1 and OMe, MUC5AC content in the mouse gastric tissue homogenates was significantly increased (*p* < 0.01). The OMe group showed the highest MUC5AC content, 212.50 ng/mL, which was 69.61% higher than that in the model group (*p* < 0.01). The MUC5AC content in the PFCP-2-1L group was 200.04 ng/mL, an increase of 65.66% compared to the model group (*p* < 0.01), but there was no significant difference compared to the OMe group (*p* > 0.05). Except for the PFCP-2-1H group, which was significantly lower than the NC group (*p* < 0.05), there were no significant differences between the other treatment groups and the NC group (*p* > 0.05).

As demonstrated in Figure 3J, the secretion of TGF-α in the ulcer model group was significantly lower than that in the NC group (*p* < 0.01), and the secretion of TGF-α among the PFCP-2-1 groups was significantly higher than that in the model group (*p* < 0.01 or *p* < 0.05). The secretion of TGF-α in the PFCP-2-1M and PFCP-2-1L groups was 499.14 pg/mL and 635.42 pg/mL, which increased by 87.74% and 139.00% respectively compared with the model group, with a significant difference (*p* < 0.01). Among them, the group with the best preventive effect was the PFCP-2-1L group, which was better than the OMe group, but there was no significant difference between them (*p* > 0.05).

#### 2.2.4. Effects on Tight Junction Protein Expression

**Figure 4:**
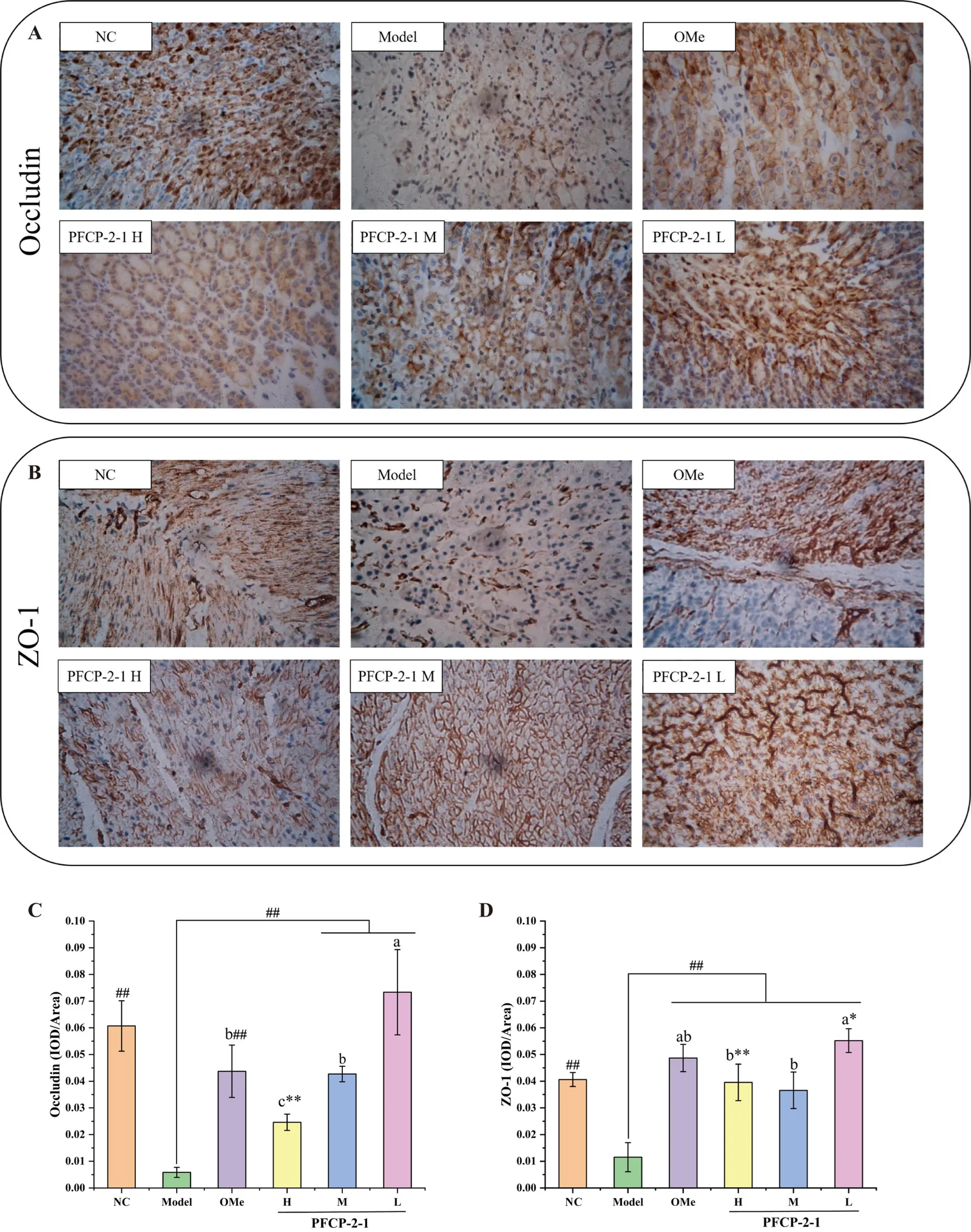
(**A**) Expression of tight junction protein Occludin in mouse gastric mucosa; (**B**) expression of tight junction protein ZO-1 in mouse gastric mucosa; (**C**) IOD/Area value of Occludin immunohistochemical results in mouse gastric mucosa; (**D**) IOD/Area value of ZO-1 immunohistochemical results in mouse gastric mucosa. Different letters indicate significant differences, *p* < 0.05; Versus NC, * *p* < 0.05, ** *p* < 0.01; Versus Model, ## *p* < 0.01. NC: normal control group; Model: model group; OMe: omeprazole group; PFCP-2-1H: high-dose group of PFCP-2-1; PFCP-2-1M: medium-dose group of PFCP-2-1; PFCP-2-1L: low-dose group of PFCP-2-1.

The positive expression product of immunohistochemistry was yellow or deeper yellow brown, and the expression of the target protein could be preliminarily observed by the naked eye through the depth of immunohistochemical staining. As could be seen from Figure 4A,B, the immunohistochemical staining area in the model group was small and the color was light. Further analysis of the IOD/Area value revealed that the expression levels of Occludin and ZO-1 in the model group were significantly reduced compared to the NC group (Figure 4C,D) (*p* < 0.01). After the intervention of PFCP-2-1, the expression of Occludin was obviously regulated (Figure 4C). Notably, the expression of Occludin was significantly increased in the PFCP-2-1L and PFCP-2-1M groups (*p* < 0.01), with the PFCP-2-1L group showing better effects than the OMe group (*p* < 0.05). Interestingly, similar to the changes in Occludin expression, PFCP-2-1 treatment also significantly regulated the expression of ZO-1 (Figure 4D). Compared with the model group, the expression of ZO-1 in gastric tissue was significantly increased after PFCP-2-1 treatment and positive drug omeprazole treatment (*p* < 0.01). The effect of upregulating ZO-1 expression in the PFCP-2-1L group was the best, and there was no significant difference between the PFCP-2-1L group and OMe group (*p* > 0.05).

#### 2.2.5. Effects on Gut Microbiota Composition

**Figure 5:**
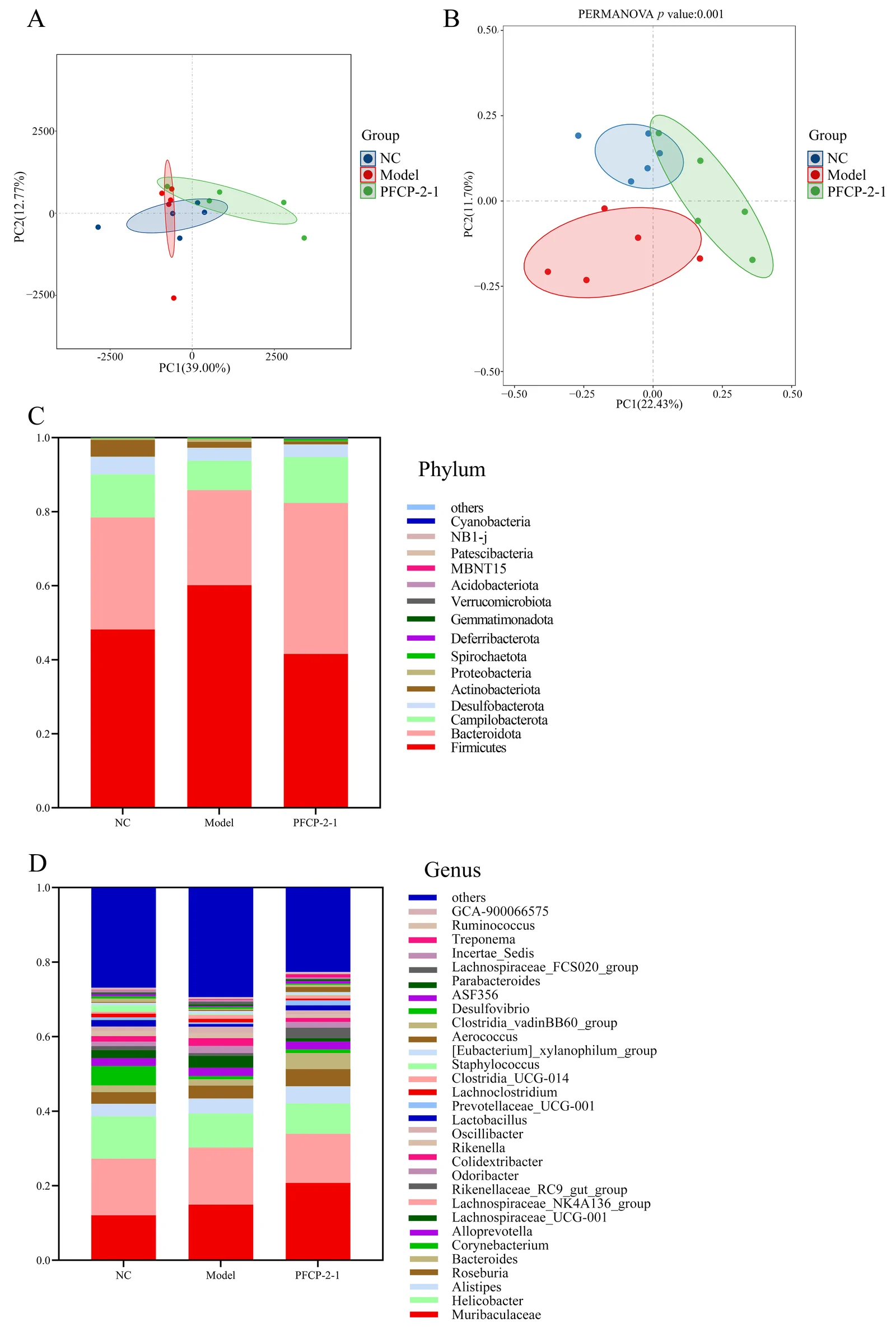
(**A**) Results of PCA analysis; (**B**) results of PCoA analysis; (**C**) bar chart of relative abundance at the phylum level (Top 30); (**D**) bar chart of relative abundance at the genus level (Top 30). NC: normal control group; Model: model group; PFCP-2-1: polysaccharide-2-1 of Finger Citron-pickled products.

As illustrated in Figure 5A, the results of beta diversity analysis show that the cumulative contribution rate of the two principal components was 51.77%, and the model group, NC group and PFCP-2-1L group all had overlapping parts, but the principal component 1 (PC1) of the PFCP-2-1 group was obviously different from that of the model group, and, in PCA analysis, the model group and NC group had more overlapping parts. From Figure 5B, it was PCoA analysis. There was no overlap between the NC group and PFCP-2-1L group in Figure 5B, but PFCP-2-1L and the model group still had some overlap. Based on the above results, it was suggested that acute injury with ethanol for 1 h might not change the intestinal flora structure of mice much.

As shown in Figure 5C, at the phylum level, the dominant groups of mice in each group were Firmicutes, Bacteroidetes, Campylobacterota, Desulfobacterota, Actinobacteriota, Proteobacteria, and so on. Compared with the NC group, the relative abundance of Firmicutes in the model group was significantly higher than that in the NC group, Bacteroidetes was lower than that in the NC group, and the ratio of Firmicutes to Bacteroidetes (F/B) increased. However, after the intervention of PFCP-2-1, the abundance of Firmicutes in mice decreased significantly, and the abundance of Bacteroidetes increased significantly.

As presented in Figure 5D, at the genus level, the dominant bacteria of mice in each group were Muribaculaceae, Lachnospiraceae_NK4A136-Group, *Helicobacter*, *Alistipes*, *Roseburia*, *Bacteroides*, *Corynebacterium*, Lachnospiraceae_UCG-001 and *Rikenella*ceae_RC9_gut_group, etc. The results showed that the relative abundance of *Lactobacillus* and *Rikenella*ceae_RC9_gut_group in the cecum of mice in the model group decreased obviously after acute induction with ethanol, and the abundance of these two bacteria could be increased after the intervention of PFCP-2-1. In addition, after the intervention of PFCP-2-1L, the relative abundance of *Helicobacter*, *Rikenella* and *Colidextribacter* decreased significantly, while *Bacteroides*, *Prevotella*ceae_UCG-001, *Ruminococcus* and *Treponema* increased significantly. The above results indicated that PFCP-2-1L intervention decreased the flora abundance related to inflammation, gastric cancer and other diseases in the intestinal flora of mice with gastric ulcer at the level of phylum and genus, while increasing the abundance of the probiotic *Prevotella*, which promoted SCFA secretion. Moreover, PFCP-2-1 reversed the increase in Firmicutes/Bacteroidetes ratio caused by acute gastric ulcer, improved the imbalance of intestinal flora and maintained intestinal homeostasis.

#### 2.2.6. Effects on SCFAs

The contents of acetic acid, propionic acid, isobutyric acid, butyric acid and valeric acid in mouse cecum were detected by gas chromatography. As displayed in Table 3, there was no significant difference between the model group and the NC group in SCFA content (*p* > 0.05). However, the contents of acetic acid and butyric acid in the PFCP-2-1L group were significantly higher than those in the NC group and model group (*p* < 0.05). The content of valeric acid was significantly higher than that of the model group (*p* < 0.05). Intervention with PFCP-2-1L showed a slight increase in propionic acid and isobutyric acid, but there was no significant difference among the three groups (*p* > 0.05). The above results indicated that PFCP-2-1 can effectively increase the contents of acetic acid, butyric acid and valeric acid.

#### 2.2.7. Correlation Analysis

**Figure 6:**
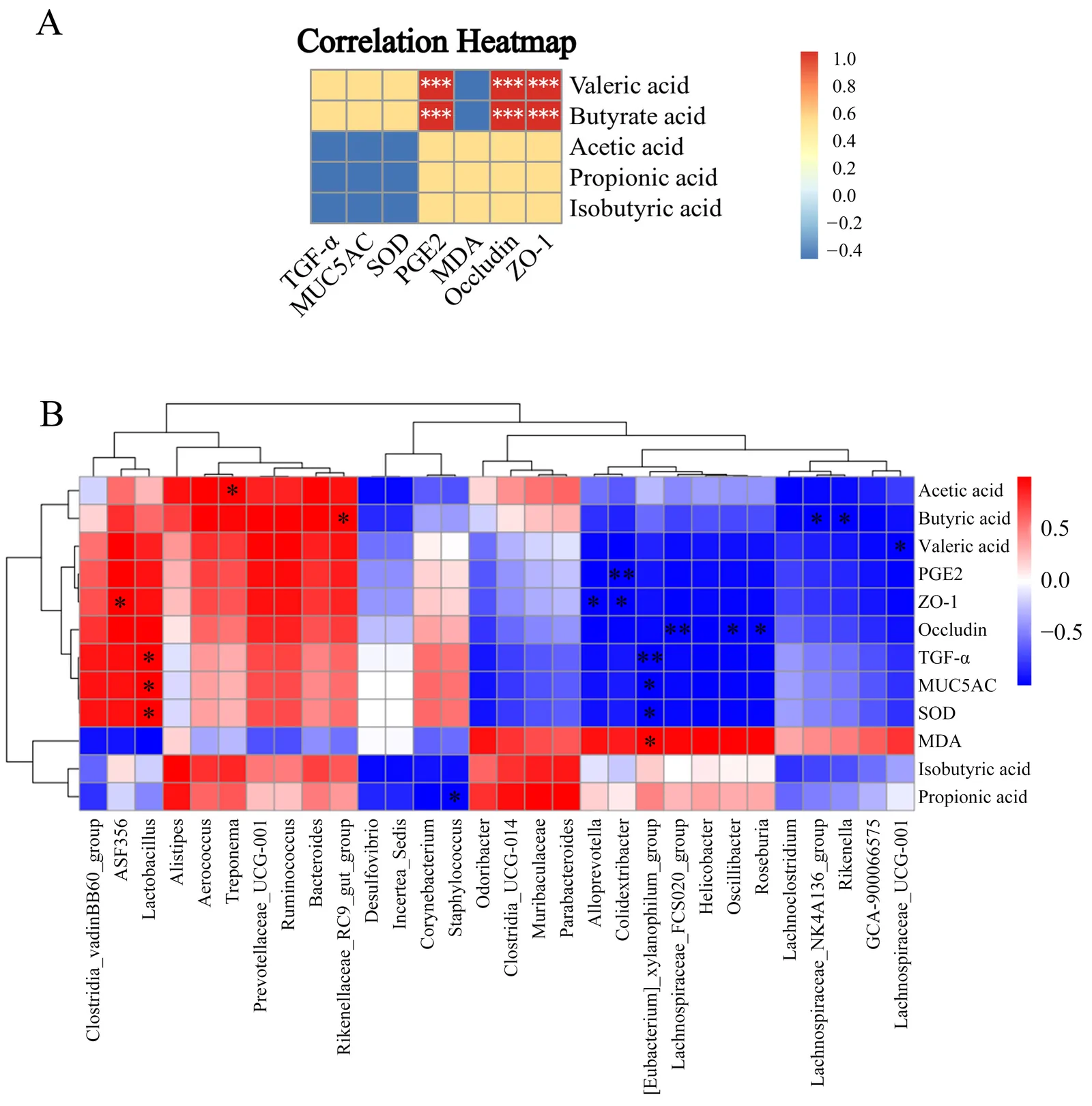
(**A**) Heatmap of Pearson correlation analysis between biochemical indices and SCFAs in mice; (**B**) heatmap of Pearson correlation analysis between biochemical indices, SCFAs, and gut microbiota in mice. Different letters indicate significant differences, *p* < 0.05; Versus NC, * *p* < 0.05, ** *p* < 0.01, *** *p* < 0.001.

As demonstrated in Figure 6A, it can be seen from the figure that the gastric mucosal protective factor PGE2 was significantly positively correlated with valeric acid and butyric acid. Figure 6B shows the correlation analysis of 30 genera of flora with SCFAs and biochemical indexes. The correlation analysis between 30 flora genera and SCFA showed that the change in acetic acid content was positively correlated with *Treponema*. Propionic acid content was negatively correlated with *Staphylococcus*. The variation in butyric acid content was positively correlated with *Rikenella*ceae_RC9_gut_group, and negatively correlated with Lachnospiraceae_NK4A136_group and *Rikenella*. The change in valeric acid content was negatively correlated with Lachnospiraceae_UCG-001. The correlation analysis between 30 flora genera and biochemical indexes showed that PGE2 in mouse stomach tissue was negatively correlated with *Colidextribacter*. ZO-1, a tight junction protein, was negatively correlated with *Colidextribacter* and *Alloprevotella*, and positively correlated with ASF356. Occludin was negatively correlated with *Roseburia*, *Oscillibacter*, and Lachnospiraceae_FCS020_group. TGF-α and MUC5AC were negatively correlated with *[Eubacterium]_xylanophilum_group*, and positively correlated with *Lactobacillus*. In this study, the contents of *Colidextribacter*, *Alloprevotella*, *Roseburia*, *Oscillibacter* and Lachnospiraceae_FCS020_group in the cecum of mice in the model group showed an obvious upward trend, while the correlation analysis showed that PGE2, tight junction protein ZO-1 and Occludin were negatively correlated with these flora. Furthermore, it could be seen from Figure 6A that the levels of ZO-1, Occludin, and PGE2 were significantly positively correlated with valeric acid and butyric acid.

## 3. Discussion

In this study, a novel acidic polysaccharide, PFCP-2-1, was purified from Laoxianghuang, and its gastroprotective effects were evaluated in an ethanol-induced acute gastric ulcer model. The results demonstrated that PFCP-2-1 could protect against ethanol-induced acute gastric mucosal injury, as evidenced by reduced ulcer area, restored mucosal architecture, enhanced antioxidant capacity, and increased levels of mucosal defense factors, which were consistent with previous studies on polysaccharides from various natural sources. For instance, the Laoxianghuang polysaccharide has been reported to effectively protect the structural integrity of gastric mucosa and achieve the effect of preventing gastric ulcer, and this result was similar to the study of Echinacea purpurea and Horse chestnut, in which the extracted polysaccharides intervened in ethanol-injured mice with a significant decrease in the ulcer index, and both of them were effective in preventing ethanol-induced gastric mucosal damage [12,13]. The pathological sections showed that the polysaccharide of Laoxianghuang had a certain preventive and protective effect on the gastric mucosa of mice with ethanol-induced acute gastric ulcer, which was consistent in the research of many plant polysaccharides, such as Codonopsis pilosula root polysaccharide [14], Momordica charantia polysaccharide [15] and Dendrobium officinale polysaccharide [16]. Furthermore, we had previously confirmed in in vitro cell experiments that the intervention of Laoxianghuang polysaccharide could enhance the SOD activity of GES-1 cells [9], reduce the secretion of MDA, and alleviate the excessive oxidative stress of cells after ethanol injury, which was consistent with the in vivo results of the present study. Combining the results of gastric ulcer area and pathological changes in acute gastric ulcer mice after intervention with Laoxianghuang polysaccharides indicated that PFCP-2-1 could exert the protective activity of gastric mucosa by enhancing the antioxidant capacity of the body in vivo.

Regarding the structural characteristics, PFCP-2-1 was identified as a high-molecular-weight (749.38 kDa) acidic heteropolysaccharide rich in galacturonic acid and galactose (molar ratio 0.147:0.238:0.059:0.556), which is broadly consistent with the compositional profile previously reported for Laoxianghuang polysaccharides [17]. Such a high molecular weight may contribute to gastroprotective activity, as similarly high-molecular-weight polysaccharides from cactus and *Hericium erinaceus* have exhibited greater mucosal protection than their low-molecular-weight counterparts [18,19,20]. The abundance of galacturonic acid, together with the presence of galactose, glucose and rhamnose, aligns with the typical monosaccharide composition of gastroprotective acidic polysaccharides [2,21].

FT-IR spectroscopy confirmed the expected pyranose ring structure and the coexistence of α- and β-anomeric configurations [22,23,24,25,26,27]. Methylation analyses identified 16 types of glycosidic linkages, predominantly derived from galactose residues; the absence of uronic acid-derived signals can be attributed to β-elimination under the alkaline conditions used, as the carboxyl groups were not reduced prior to methylation. Combining 1D and 2D NMR analyses (Table 2, Figure 2) established the backbone as →4)-α-D-GalpA-(1→6,4)-α-D-Galp-(1→[→3)-β-D-Glcp-(1]_n_[→4)-α-D-GalpA6Me-(1]_p_[→3)-α-D-Rhap-(1]_m_→, with branches possibly attached via (1→4) linkages at the galactose residue, in agreement with previously reported pectic-type polysaccharide structures [28,29,30]. Although the high molecular weight and limited solubility somewhat reduced the signal-to-noise ratio in certain 2D NMR regions, the key correlations supporting the proposed structure were clearly observable and were corroborated by methylation and monosaccharide composition data. It is noteworthy that the gastroprotective efficacy of polysaccharides is not uniform, but varies with the ulcer-inducing agent, reflecting the divergent pathogenic mechanisms underlying ethanol and nonsteroidal anti-inflammatory drug (NSAID)-induced gastric injury [31]. Ethanol-induced damage exhibits a well-documented concentration-dependent profile, with concentrations exceeding 10–15% (*v*/*v*) disrupting the gastric mucosal barrier in humans, and higher concentrations (>10%) causing lesions that require over 24 h for resolution, primarily mediated by oxidative stress and microvascular injury [32,33,34]. In contrast, NSAIDs such as indomethacin exert their ulcerogenic effects mainly through cyclooxygenase inhibition, leading to reduced prostaglandin synthesis, alongside neutrophil infiltration [35]. Despite these mechanistic distinctions, several polysaccharides have demonstrated protective activity against both types of lesions; for example, *Angelica sinensis* polysaccharides dose-dependently attenuated gastric damage induced by either ethanol or indomethacin in rats [35]. Nevertheless, whether PFCP-2-1 confers comparable protection in NSAID-induced gastric injury models warrants further investigation.

Regarding the gastroprotective mechanisms, excessive oxidative stress was an important cause of gastric mucosal injury [36]. SOD activity and MDA content were important indicators to measure the antioxidant capacity of the body, which could reflect the rate and intensity of oxidative stress of the body and indirectly reflect the degree of peroxide damage of tissues [14]. The results showed that the Laoxianghuang polysaccharides could increase SOD enzyme activity and enhance the antioxidant capacity in mice, which might help alleviate ethanol-induced gastric mucosal damage. Ethanol-induced injury would increase the lipid peroxidation of the body and cause oxidative stress injury [37]. However, both PFCP-2-1 and omeprazole interventions effectively reduced the serum MDA levels in mice with ethanol-induced acute gastric injury. It showed that the low dose of polysaccharide from Laoxianghuang can reduce the MDA value in the serum of mice, thus achieving the effect of preventing oxidation. PGE2 was a defensive factor of gastric mucosa, which played a key role in the defense of gastric mucosa and could maintain the integrity of mucosal cells by improving blood flow [38,39]. The results demonstrated that PFCP-2-1 may strengthen the protective barrier of gastric mucosa by increasing the content of PGE2, a protective factor of gastric mucosa in mice. MUC5AC is the most abundant mucin subtype expressed in gastric tissue, which had a vital influence on maintaining the gastric mucus barrier [40]. It had been found that most natural polysaccharides could promote the expression of MUC5AC mucin, thus protecting the mucous layer in the stomach tissue of rats or mice [40]. The results indicated that alcohol exposure caused damage to the gastric mucosal barrier and reduced MUC5AC secretion, while PFCP-2-1 intervention could increase MUC5AC levels in gastric tissue, strengthen the gastric mucus barrier, and reduce ethanol-induced gastric mucosal injury. TGF-α is an important defense factor of gastric mucosa, which is mainly synthesized by its own mucosal cells, and its expression level was the highest in gastric antrum mucosa [41]. When the gastric mucosa was stimulated and damaged, the expression of TGF-α increases, which accelerated the migration of differentiated epidermal cells to granulation tissue, thereby reconstructing the mucosal structure [21]. The intervention of the PFCP-2-1 can increase the secretion of TGF-α in gastric tissue, thus speeding up the mucosal reconstruction when gastric mucosa was stimulated and damaged, and achieving the effect of protecting gastric mucosa.

Occludin and ZO-1 were two important tight junction proteins, which not only participate in the regulation of cell material transport and maintain the tissue and integrity of epithelial tight junction, but also were closely related to the process of cell proliferation, differentiation and migration. Once their expression was destroyed, the tight junction structure and function of gastric epidermis will also change, eventually leading to the loss of the gastric epithelial barrier and causing it to be easy to damage by external stimuli [42]. The mechanical barrier of gastric mucosa was mainly composed of intercellular junction complex and mucosal epithelial cells, while the intercellular junction complex of mucosal epithelial cells was composed of tight junction, adhesive junction, desmosome and gap junction from top to bottom. Tight connection can make the cell membranes of different cells closely contact each other, leaving almost no gaps, thus forming a physiological barrier, effectively preventing the attack of external stimuli [43]. Tight junction protein played an important role in maintaining the structural integrity of gastric mucosa and repairing the early superficial injury of gastric mucosa. When the gastric mucosa was shallowly injured, the necrotic cells on the surface of the injured part fall off, while the healthy cells at the upper end of the gastric gland could extend their pseudopods to migrate to the surface, forming tight junctions between cells until the injured area was completely covered and the integrity of the mucosa was restored [44]. The above results indicated that oral administration of PFCP-2-1 could effectively improve the expression of tight junction protein in gastric mucosa and maintain the stability and integrity of the mucosal mechanical barrier.

It should be noted, however, that a most intriguing and concerning finding is the non-linear dose–response relationship, where the lowest dose of PFCP-2-1 (25 mg/kg) consistently outperformed the middle (50 mg/kg) and high (100 mg/kg) doses across nearly all measured parameters, including ulcer index, SOD, MDA, PGE2, MUC5AC, and tight junction markers. This phenomenon is contrary to the conventional pharmacological expectation of a dose-dependent positive effect [45], and warrants in-depth discussion. We propose several non-mutually exclusive hypotheses for this inverse dose–response. Firstly, the high molecular weight of PFCP-2-1 (approximately 749.38 kDa) may lead to significantly increased viscosity and molecular aggregation at higher concentrations. Studies have shown that high-molecular-weight polysaccharides generally exhibit high viscosity and low solubility, which can slow down their transport and absorption efficiency in the gastrointestinal tract, thereby limiting the full expression of their biological activity [46]. It is thus plausible that PFCP-2-1 at high doses, due to a sharp rise in solution viscosity and intensified molecular aggregation, may experience reduced efficiency in interacting with the gastric mucosa, consequently weakening its protective effects. Secondly, high doses of macromolecular polysaccharides may induce regulatory responses that counteract a further enhancement of protection. For instance, high-dose Astragalus polysaccharides have been reported to promote excessive Th17 cell responses, which in certain pathological contexts may shift the immune balance without necessarily providing additional benefit [47]. In the present study, the PFCP-2-1H group exhibited a lower SOD activity (165.05 U/mL) and a higher MDA content (11.77 nmol/mL) compared to the low-dose group (179.41 U/mL and 9.18 nmol/mL, respectively), indicating that the activation of the antioxidant defense system was not proportionally strengthened, but was instead attenuated at the highest dose. Thirdly, the hormesis hypothesis may partially explain the dose–response pattern observed in this study. Hormesis describes a biphasic response in which low-dose exposure elicits beneficial effects, whereas high doses may lead to inhibitory or suboptimal outcomes [48]. Although hormesis is more commonly studied in the context of toxins and stressors, recent investigations have extended this concept to the immunomodulatory field of biological macromolecules, including polysaccharides. Zhang et al. reported the hormetic effect of a natural polysaccharide for the first time, the pectic polysaccharide AELP-B6 from *Acanthopanax senticosus*, which promoted immune factor release and drove M1-like macrophage polarization at low and medium doses, while, at high doses, it enhanced the expression of apoptosis-related proteins and activated the endogenous apoptotic cascade, suggesting that excessive stimulation may engage negative-feedback regulation [49]. PFCP-2-1, as a pectin-type polysaccharide rich in galacturonic acid, may, at low doses, serve as a mild stress signal that moderately activates gastric mucosal defense mechanisms; however, when the dose is too high, this stimulus may exceed the optimal response range of the organism, instead triggering inhibitory regulation. The specific mechanisms underlying the diminished protective efficacy of PFCP-2-1 at high doses warrant further validation through pharmacokinetic experiments.

Regarding the intestinal flora regulation, beta diversity analysis showed that the model group and NC group had more overlapping parts, which might be due to the fact that the gut microbiota structure had not yet been significantly altered at one hour after ethanol administration. Based on the above results, it was suggested that acute injury with ethanol for 1 h might not change the intestinal flora structure of mice much. However, the intervention with PFCP-2-1L showed that the intestinal flora had formed a diverse and characteristic flora which was different from the model group and NC group. This might be that the intervention of PFCP-2-1 had promoted the growth of some flora, which also reflected the regulation of polysaccharide on the intestinal flora to some extent. Usually, the digestive enzymes secreted by the body could only break down a few polysaccharides, while most of them had complex chemical structures or special bonding patterns, such as β-glycosidic linkages, and could not be directly absorbed and utilized [50,51]. When the human body ingested polysaccharides, most of the polysaccharides entered the large intestine without being digested, where they then interacted with the intestinal microflora. It had been found that intestinal microflora played an important role in initiating or maintaining intestinal immunity in inflammatory reactions by providing antigens or other stimulating factors closely related to gastrointestinal diseases, so the homeostasis of intestinal microflora was a key factor affecting gastrointestinal health [52]. Notably, remodeling intestinal flora through diet (such as functional active substances such as polysaccharides) or other means has been proved to have beneficial effects in maintaining the functional integrity of intestinal tract and alleviating gastric mucosal injury [53]. At the phylum level, the relative abundance of Firmicutes in the model group was significantly higher than that in the NC group, Bacteroidetes was lower than that in the NC group, and the ratio of Firmicutes to Bacteroidetes (F/B) increased, indicating that ethanol-induced gastric mucosal damage might disrupt the balance between these two phyla of the intestinal flora. However, after the intervention of PFCP-2-1L, the abundance of Firmicutes in mice decreased significantly, and the abundance of *Bacteroides* increased significantly. The results were similar to the study by Yang et al. [54], who found that ethanol would lead to a decrease in the abundance of Bacteroidetes, which was moderated when treated with α-mangostin, with a decrease in F/B and an improvement in gastric ulceration in rats. In addition, Firmicutes/Bacteroidetes ratio was associated with inflammatory diseases and gastric mucosal damage [55,56], which might be exacerbated when F/B is elevated, delaying the healing of ulcers. Therefore, interventions that limit the increase in Firmicutes or promote the abundance of Bacteroidetes could help in the treatment of gastric ulcers.

At the genus level, the results showed that the relative abundance of *Lactobacillus* and *Rikenella*ceae_RC9_gut_group in the cecum of mice in the model group decreased obviously after acute induction with ethanol, and the abundance of these two bacteria could be increased after the intervention of PFCP-2-1. Ethanol intake led to impaired intestinal barrier function, mucosal inflammation, and oxidative stress, thereby inhibiting the growth of beneficial bacteria and promoting the proliferation of opportunistic pathogens [57]. The reduction in *Lactobacillus* might have been related to ethanol-induced changes in the intestinal acidic environment and increased nutritional competition, as this genus was relatively sensitive to environmental fluctuations [58]. Similarly, the decrease in *Rikenella*ceae_RC9_gut_group might have resulted from ethanol-induced disturbances in bile acid metabolism and inflammatory responses, since this bacterial group played a crucial role in maintaining intestinal homeostasis [57]. PFCP-2-1L treatment alleviated these phenomena mainly due to its prebiotic properties and regulatory effects on the gut microecology. Specifically, the polysaccharide acted as a prebiotic substrate fermented by intestinal microbiota to produce SCFAs, which lowered intestinal pH and created an environment favorable for the growth of beneficial bacteria such as *Lactobacillus* [59]. Moreover, the polysaccharide mitigated ethanol-induced intestinal inflammation and oxidative stress, thereby improving the dysbiosis of gut microbiota [60]. For instance, polysaccharide components in camel milk regulated the IL-17 and TNF-α signaling pathways to reduce inflammation and promote the restoration of *Lactobacillus* populations [57]. In addition, after the intervention of PFCP-2-1L, the relative abundance of *Helicobacter*, *Rikenella* and *Colidextribacter* decreased significantly, while *Bacteroides*, *Prevotella*ceae_UCG-001, *Ruminococcus* and *Treponema* increased significantly. Polysaccharide supplementation also modulated specific bacterial taxa associated with gut health and host metabolism. It promoted the proliferation of *Bacteroides*, which fermented polysaccharides to generate SCFAs, thereby strengthening the intestinal barrier and reducing endotoxin translocation [61]. In addition, the abundance of *Prevotella*ceae UCG-001 increased, as this bacterium participated in the degradation of cellulose and xylan, contributing to the improvement in glucose and lipid metabolic disorders [62]. Furthermore, the abundances of *Ruminococcus* and *Treponema* were elevated following polysaccharide intervention; both genera fermented dietary fibers to produce SCFAs (e.g., butyrate), which helped suppress hepatic inflammation and oxidative stress [63]. The above results indicated that PFCP-2-1 decreased the flora abundance related to inflammation, gastric cancer and other diseases in the intestinal flora of mice with gastric ulcers at the levels of phylum and genus, while increasing the abundance of the probiotic *Prevotella*, which promoted SCFA secretion [64]. Moreover, PFCP-2-1 reversed the increase in the Firmicutes/Bacteroidetes ratio caused by acute gastric ulcer, improved the imbalance of intestinal flora and maintained intestinal homeostasis.

SCFAs, as an important energy source of intestinal microflora and host, played an important role in human health and diseases through different modes of action [65]. As one of the components of SCFAs, butyrate was considered a key energy source, which helped to maintain the intestinal barrier and protect the host from potential pathogens entering the gastrointestinal tract [66]. The results showed that there was no significant difference between the model group and the NC group in SCFA content (*p* > 0.05), which might be due to the fact that the early feeding and gavage methods of the model group were consistent with those of the NC group, so the SCFA content in the body had not changed much. However, the contents of acetic acid and butyric acid in mice were significantly higher than those in the NC group and model group (*p* < 0.05). The content of valeric acid was significantly higher than that of the model group (*p* < 0.05). Intervention with PFCP-2-1 showed a slight increase in propionic acid and isobutyric acid, but there was no significant difference among the three groups (*p* > 0.05). The above results indicated that PFCP-2-1L can effectively increase the contents of acetic acid, butyric acid and valeric acid, which may be associated with its gastric mucosal protective effects.

Correlation analysis was a method in statistics to measure the linear relationship and strength between variables. In biomedical research, correlation analysis was commonly used to reveal the relationship between biomolecules such as genes, proteins, metabolites, etc., and disease phenotypes, clinical indicators, or environmental factors, e.g., correlation analysis of intestinal flora with host metabolism and inflammatory factors [67]. The correlation analysis between 30 flora genera and SCFA showed that the change in acetic acid content was positively correlated with *Treponema*. Propionic acid content was negatively correlated with *Staphylococcus*. The variation in butyric acid content was positively correlated with the *Rikenella*ceae_RC9_gut_group, and negatively correlated with Lachnospiraceae_NK4A136_group and *Rikenella*. The change in valeric acid content was negatively correlated with Lachnospiraceae_UCG-001, indicating that the production of SCFAs was influenced by many intestinal bacteria. Cui et al. [68] found that Scutellaria baicalensis Georgi polysaccharide could decrease the abundance of *Staphylococcus* and increase the content of propionic acid, which was consistent with Pearson correlation analysis in this study, and *Staphylococcus* was negatively correlated with the increase in propionic acid content. The correlation analysis between 30 flora genera and biochemical indexes showed that PGE2 in mouse stomach tissue was negatively correlated with *Colidextribacter*. ZO-1, a tight junction protein, was negatively correlated with *Colidextribacter* and *Alloprevotella*, and positively correlated with ASF356. Occludin was negatively correlated with *Roseburia*, *Oscillibacter*, and Lachnospiraceae_FCS020_group. TGF-α and MUC5AC were negatively correlated with *[Eubacterium]_xylanophilum_group*, and positively correlated with *Lactobacillus*. In this study, the contents of *Colidextribacter*, *Alloprevotella*, *Roseburia*, *Oscillibacter* and Lachnospiraceae_FCS020_group in the cecum of mice in the model group showed an obvious upward trend, while the correlation analysis showed that PGE2, tight junction protein ZO-1 and Occludin were negatively correlated with these flora. Furthermore, it can be seen from Figure 6A that the contents of tight junction protein ZO-1, Occludin and PGE2 were significantly positively correlated with valeric acid and butyric acid. These results suggest that PFCP-2-1 induced modulation of gut microbiota composition and SCFA levels is associated with increased expression of ZO-1, Occludin and PGE2, which may contribute to the maintenance of gastric epithelial barrier function and be associated with the observed gastric mucosal protective effects. This potential mechanism may help maintain epithelial tight junctions and tissue integrity [69]. However, the elevation in MUC5AC and TGF-α, induced by PFCP-2-1, might not be mediated through the regulation of gut microbiota. Instead, it was likely attributed to its interaction with cell surface receptors, such as Toll-like receptors (TLRs), which activated downstream signaling pathways to promote TGF-α expression [70]. Subsequently, TGF-α might act through autocrine or paracrine mechanisms on epidermal growth factor receptors (EGFRs), activating the EGFR signaling pathway and ultimately upregulating the expression of MUC5AC [70]. It should be noted that the observed associations between SCFAs and gastric mucosal barrier indicators are correlative, and further mechanistic studies are required to clarify their causal relationships. However, it is also important to recognize that our microbiota data were only collected from the PFCP-2-1L, while the middle-dose, high-dose, and positive drug control groups were missing, which makes it impossible to quantify dose-dependent relationships and demonstrate the differences or advantages of this polysaccharide and the positive control in regulating gut microbiota. Furthermore, the SCFA data do not support strong mechanistic claims, as Table 3 shows no significant differences in SCFAs between the NC group and the model group, while PFCP-2-1 increased acetic acid, butyric acid, and valeric acid.

Beyond the polysaccharide itself, other bioactive constituents inherent to the fermented preparation, such as phenolic compounds and organic acids, may also contribute synergistically to the observed gastroprotective effects. Laoxianghuang is produced through a complex fermentation process involving multiple microbial and enzymatic transformations, which can generate or release a variety of secondary metabolites. Although the present study focused exclusively on the purified polysaccharide fraction, it is plausible that the presence of these low-molecular-weight compounds in the crude extract could complement or potentiate the activity of PFCP-2-1 in the context of the whole food matrix. This potential synergy warrants further investigation in future studies.

On the other hand, the structure–activity relationship of gastroprotective polysaccharides is complex and multifactorial. According to previous reviews on gastroprotective polysaccharides, their structure–activity relationships are complex and multifactorial [2]. In general, both high- and low-molecular-weight polysaccharides have been reported to exhibit gastroprotective activity, depending on their physicochemical properties and evaluation models. Structural features such as the presence of uronic acids, especially galacturonic acid, monosaccharide composition including Gal and Ara, backbone composition, degree of branching, and polysaccharide conformation have all been suggested to contribute to gastric mucosal protection. In addition, functional groups such as sulfate or acetyl substituents, as well as the association with phenolic compounds, may further influence biological activity. In the present study, PFCP-2-1 is a high-molecular-weight acidic polysaccharide rich in galacturonic acid and galactose, with a pectic-type backbone structure. These characteristics share similarities with structural features reported for other gastroprotective polysaccharides in the literature. However, it should be noted that the present work does not aim to establish a direct structure–activity relationship. Instead, the structural characteristics of PFCP-2-1 are discussed in the context of existing studies, and further comparative and mechanistic investigations are required to clarify how specific structural elements may contribute to its gastroprotective effects.

## 4. Materials and Methods

### 4.1. Materials and Reagents

Laoxianghuang was provided by Guangzhou Zhancui Food Co., Ltd. (Guangzhou, China); DEAE-52 cellulose filler and agarose CL-6B gel filler were provided by Beijing Ruida Henghui Technology Development Co., Ltd. (Beijing, China); 3500 MW dialysis bag was provided by Guangzhou Qiyun Zuo Ke Biotechnology Co., Ltd. (Guangzhou, China); MUC5AC, PGE2 and TGF-α ELISA kits were purchased from Wuhan Huamei Bioengineering Co., Ltd. (Wuhan, China); ZO-1 and Occludin were purchased from Abcam Antibody Company (Cambridge, UK); H&E dyeing set was purchased from Biossci company (Wuhan, China); acetic acid, propionic acid, butyric acid, isobutyric acid, valeric acid, and isovaleric acid were purchased from McLean Reagent Company (Shanghai, China). SPF KM mice, weighing 20 ± 2 g, were provided by the Experimental Animal Center of Southern Medical University (Guangzhou, China).

### 4.2. Sample Preparation

The extraction and purification of Laoxianghuang polysaccharides were performed according to a previously reported method with minor modifications [9]. Briefly, dried Laoxianghuang powder (passed through a 30-mesh sieve) was extracted twice with distilled water at a ratio of 1:31 (g/mL) at 90 °C for 1.5 h each. The combined filtrates were concentrated, mixed with five volumes of 95% ethanol, and stored at 4 °C overnight to obtain the crude polysaccharide. To remove proteins, the crude polysaccharide was subjected to the Sevag method (chloroform: n-butanol = 4:1, *v*/*v*) for five repetitions until the absorbance at 280 nm was negligible. The deproteinized solution was dialyzed (3500 MW cutoff) and lyophilized to obtain PFCP. PFCP was then fractionated on a DEAE-52 cellulose column eluted with a stepwise gradient of NaCl (0, 0.1, 0.3, and 0.5 M) at a flow rate of 1.0 mL/min. The major polysaccharide fraction (PFCP-2) eluted with 0.3 M NaCl was further purified on a CL-6B agarose gel column. The main peak (PFCP-2-1) was collected, dialyzed, and lyophilized. The pH of all solutions was adjusted to 7.0 prior to column chromatography.

### 4.3. Characterization of PFCP-2-1

#### 4.3.1. SEM Analysis

PFCP-2-1 was affixed to a sample platform lined with double-sided tape, then slightly pressed with a capillary to embed some of the sample into the tape; after removing the surface material with a blade and blowing off the surplus polysaccharide using an air duster, it was transferred to an ion sputter instrument, where gold was sprayed under a 10 mA current to coat the surface with a platinum film, and its morphology was subsequently examined using an EVO MA 15 scanning electron microscope (ZEISS, Oberkochen, Germany) at an 8.0 kV accelerating voltage [71].

#### 4.3.2. AFM Analysis

The PFCP-2-1 components were prepared as 10 μg/mL solutions in distilled water, and 5 μL of each solution was dropped onto a mica sheet; the sample was dried at room temperature in a desiccator and subsequently examined using a Multimode 8 atomic force microscope (Bruker, Billerica, MA, USA). The test was carried out at room temperature and atmosphere, with a scanning range of 5.0 × 5.0 μm and a scanning frequency of 1.00 Hz [72].

#### 4.3.3. Molecular Weight Detection

PFCP-2-1 was detected by high-performance gel permeation chromatography [72]. A series of pullulan standard samples with different concentrations, along with the sample, were dissolved in distilled water. After passing the solution through a 0.22 µm filter membrane, it was loaded for analysis. A high-performance liquid chromatography system, outfitted with a differential refractive index detector (Shimadzu, Kyoto, Japan) and connected in series with TSKgel G5000 PWxl and TSKgel G3000 PWxl gel columns, was employed. A 20 μL sample was injected into the system, eluted with pure water at a flow rate of 0.6 mL/min, while the column compartment was kept at 35 °C. A standard curve for the logarithm of the relative molecular weight (LgMw) versus the elution volume (V) was constructed: lgMW = 0.0213 V_2_ − 1.0581 V + 15.996.

#### 4.3.4. Monosaccharide Composition Analysis

Monosaccharide composition was analyzed using an ICS5000 ion chromatography system (Thermo Fisher, Waltham, MA, USA) equipped with a CarboPac PA20 column (3 × 150 mm) [73]. Briefly, 5 mg of PFCP-2-1 was hydrolyzed with 2 mL of 3 M TFA at 120 °C for 3 h. The hydrolysate was dried under nitrogen, reconstituted in 5 mL of water, and diluted 20-fold prior to injection (5 µL). The mobile phases consisted of A: H_2_O; B: 15 mM NaOH; and C: 15 mM NaOH + 100 mM NaOAc, with a flow rate of 0.3 mL/min at 30 °C. Quantification was performed using an external standard method, with calibration curves (peak area vs. concentration) established for each monosaccharide standard (R^2^ > 0.995). All analyses were performed in triplicate.

#### 4.3.5. FT-IR Analysis

An accurate 2.0 mg of dried PFCP-2-1 was weighed and thoroughly mixed with 200.0 mg of dried potassium bromide in an agate mortar, then uniformly ground under an infrared lamp until no crystalline particles were observed, after which it was pressed into a thin pellet and placed in a desiccator. The sample was scanned using a Vertex70 FT-IR spectrometer (Bruker, Billerica, MA, USA) at a resolution of 4 cm^−1^ over a range of 4000 to 400 cm^−1^ [74].

#### 4.3.6. Methylation Analysis

Methylation analysis was performed according to the method of Ji et al. [75], with modifications. Briefly, 2-3 mg of PFCP-2-1 was dissolved in anhydrous DMSO and methylated with iodomethane in a basic solution. The permethylated product was hydrolyzed with TFA, reduced with NaBH_4_, and acetylated with acetic anhydride. The resulting partially methylated alditol acetates (PMAAs) were analyzed by GC-MS (Agilent 6890-5973, Agilent, Santa Clara, CA, USA) on an RXI-5 SIL MS column (30 m × 0.25 mm × 0.25 µm) (Restek, Bellefonte, PA, USA) with helium as the carrier gas (1 mL/min). Importantly, no carboxyl-reduction step was performed prior to methylation; consequently, the detection of uronic acid linkages was compromised, which represents a limitation of this analysis. The oven temperature was programmed from 120 °C to 250 °C at 3 °C/min and held for 5 min.

#### 4.3.7. NMR Analysis

Fifty milligrams of PFCP-2-1 were accurately weighed, vacuum-dried using P_2_O_5_ for 72 h, dissolved in 0.5 mL of D_2_O, and allowed to stand overnight; after freeze-drying, another 0.5 mL of D_2_O was added, and this cycle was repeated three times to ensure complete exchange of labile protons. The freeze-dried sample was transferred into a 5 mm NMR tube and re-dissolved in 0.5 mL of D_2_O. At 25 °C, one-dimensional (^1^H and ^13^C) as well as two-dimensional (HSQC, ^1^H−^1^H-COSY, and NOESY) NMR spectra were measured on a Bruker 500 MHz NMR spectrometer (Bruker, Rheinstetten, Germany) [20].

### 4.4. Protective Activity of PFCP-2-1 on Gastric Mucosa In Vivo

#### 4.4.1. Animal Grouping and Drug Administration Modeling

The study protocol was approved by the Ethics Committee of South China Agricultural University (approval no. SYXK 2022-0136, grant no. 2022B100, date: 7 July 2022) and was conducted under the guidance of the Laboratory Animal Center of South China Agricultural University. Specific pathogen-free (SPF) male KM mice (20 ± 2 g) were obtained from the Experimental Animal Center of Southern Medical University. All animals were allowed to acclimatize for one week. During this period, their general health status, diet, water intake, body weight, fur condition, bowel movements, and activity were monitored. A total of 60 mice were enrolled in this study.

Group assignment was performed by independent personnel who were not in-volved in subsequent experimental procedures, using a random number table method. The 60 mice were randomly divided into six groups (n = 10 per group): a normal control (NC) group, a model group, an omeprazole group (OMe, 20 mg/kg BW), a high-dose PFCP-2-1 group (PFCP-2-1H, 100 mg/kg BW), a medium-dose PFCP-2-1 group (PFCP-2-1M, 50 mg/kg BW), and a low-dose PFCP-2-1 group (PFCP-2-1L, 25 mg/kg BW).

Mice in the NC group and the model group were administered normal saline by gavage at a dose of 0.2 mL/10 g BW daily for 15 consecutive days. The OMe group and the three PFCP-2-1 dose groups received an equivalent volume of the corresponding drug suspension by gavage for the same duration. Body weights were recorded daily. On the day of the last administration, all mice were fasted for 24 h with free access to water. On the following day, animals in all groups except the NC group were administered absolute ethanol by gavage at a dose of 0.009 mL/g BW to induce gastric mucosal injury, while the NC group received an equivalent volume of saline at the same time point. One hour after modeling, mice were anesthetized with isoflurane. Blood was then drawn from the orbital sinus. Immediately after blood collection, the mice were euthanized by cervical dislocation. Subsequently, dissection was performed and tissue samples were harvested.

#### 4.4.2. Effects of PFCP-2-1 on Organ Index, Gastric Ulcer Area and Pathological Changes in Mice

After a 24 h fasting period, the mice were weighed before being sacrificed; post-euthanasia, the extracted liver, kidneys, spleen, and thymus were weighed separately, and the organ index was computed using Formula (1).

The stomach tissue was spread out, and the ulcer index was determined based on the Guth scoring criteria: the total ulcer score represented the ulcer index; pinpoint hemorrhages, erosions, and lesions less than 1 mm in length were assigned 1 point; linear hemorrhages or 1 mm lesions were given 2 points; lesions of 1–2 mm were given 3 points; 2–4 mm lesions were given 4 points; and lesions over 4 mm were given 5 points, with the score doubled if the lesion width exceeded 1 mm. The mean ulcer index for each group was determined by calculating the average score for that group. Additionally, the ulcer index was computed using Formula (2).

The stomach tissue was fixed in a 4% paraformaldehyde solution for 24 h and then processed for sectioning and subsequent examination. The procedure was adapted with minor modifications from the method reported by [71].

(1) $$\mathrm{Organ}\;\mathrm{index}\;(\mathrm{mg}/g)=\frac{M_{0}\;(\mathrm{mg})}{M_{1}\;(g)}$$

M_0_ refers to organ quality; M_1_ refers to the weight of mice.

(2) $$\mathrm{Ulcer}\;\mathrm{inhibition}\;\mathrm{rate}\;(\%)=\left(1-\frac{\mathrm{Ulcer}\;\mathrm{index}\;\mathrm{in}\;\mathrm{drug}\;\mathrm{group}}{\mathrm{Ulcer}\;\mathrm{index}\;\mathrm{in}\;\mathrm{model}\;\mathrm{group}}\right)\times 100\%$$

#### 4.4.3. Determination of Serum Indexes and Related Indexes of Gastric Homogenate in Mice

Following 15 days of continuous dosing, the animals underwent a 24 h fasting period. Blood was drawn from the mice using the orbital blood collection method. The blood was left to stand for 30 min, and then centrifuged at 5000 rpm for 10 min at 4 °C. Serum levels of the oxidative stress indicators SOD and MDA were determined following the kit protocols [76].

Stomach tissues from the mice in each group were harvested and washed with pre-chilled PBS to remove remaining blood and gastric residues, then the surface moisture was absorbed using filter paper before weighing. Physiological saline was employed as the medium for homogenization, with a tissue weight (g) to saline volume (mL) ratio of 1:9. The tissue was placed in a homogenizer and ground thoroughly to produce a 10% tissue homogenate. Subsequently, the homogenate was moved to a centrifuge tube and centrifuged at 10,000 rpm for 10 min at 4 °C, the supernatant was collected, and it was stored at −20 °C. Following that, the levels of PGE2, MUC5AC, and TGF-α were determined according to the ELISA kit protocol [19].

#### 4.4.4. Immunohistochemical Detection of ZO-1 and Occludin in Gastric Tissue

The expression of ZO-1 and Occludin was determined via immunohistochemistry, following sequential steps of antigen retrieval, endogenous peroxidase blocking, and blocking, with the resultant slides examined under a microscope [77].

#### 4.4.5. High-Throughput Sequencing of Mouse Cecum Contents

For 16S rRNA gene sequencing and SCFA quantification, cecal contents were collected from five mice per group, specifically from the normal control (NC), model, and PFCP-2-1L (25 mg/kg) groups. This selection was based on the finding that the low dose exhibited the most pronounced gastroprotective effect; however, we acknowledge this choice limits the assessment of dose-dependent microbial modulation. The samples were outsourced to Shanghai Ouyi Biomedical Technology Co., Ltd. for sequencing on an Illumina platform. Upon completion of sequencing, the data were processed and analyzed online via the proprietary Ouyi Cloud Platform (https://cloud.oebiotech.com, accessed on 25 June 2023), developed by Ouyi Biomedical to study alterations in the gut microbial structure of the mice.

#### 4.4.6. Determination of SCFAs in Mouse Cecum

SCFAs in the cecum contents of mice in the NC group, model group and PFCP-2-1 group were determined by the GC-MS method of Zhou et al. with slight modifications [78]. TSQ 8000 EVo (Thermo Fisher Scientific (China) Co., Ltd., Shanghai, China) was used with a TG-WAXMS capillary column (30 m × 0.25 mm × 0.25 μm). The samples of cecum contents in each group were mixed with methyl tert-butyl ether (MTBE), vortexed for 30 min, centrifuged at 12,000 r/min for 10 min, and then, the supernatant was taken, and an appropriate amount of anhydrous sodium sulfate was added to stand overnight. The supernatant was filtered through a 0.22 μm membrane and then analyzed. Six kinds of SCFA (acetic acid, propionic acid, butyric acid, isobutyric acid, valeric acid and isovaleric acid) standard solutions were prepared with methyl tert-butyl ether, and each SCFA standard solution was diluted into SCFA working solutions with gradient concentrations of 50, 100, 200, 400 and 800 μg/mL, and detected on the computer.

### 4.5. Statistical Analysis

Data are presented as mean ± standard deviation (SD). Statistical analyses were performed using R Programming Language (version 4.5.0). Differences among multiple groups were evaluated by one-way ANOVA followed by Tukey’s post hoc test. For correlation analyses (Pearson), the Benjamini–Hochberg false discovery rate (FDR) correction was applied to account for multiple comparisons, with an FDR threshold of q < 0.05 considered statistically significant. A *p*-value of <0.05 was considered statistically significant for ANOVA.

## 5. Conclusions

In this study, the chemical structure and in vivo gastric mucosal protective activity of PFCP-2-1, a Laoxianghuang polysaccharide purified using DEAE-52 and agarose CL-6B, were systematically analyzed. The structural analysis indicated that PFCP-2-1 was a macromolecular polysaccharide with an average molecular weight of 749.38 kDa, featuring a smooth surface and potentially forming large aggregates. It was primarily composed of rhamnose, galactose, glucose, and galacturonic acid in a molar ratio of 0.147:0.238:0.059:0.556, which demonstrated its nature as an acidic heteropolysaccharide. Further analyses using FT-IR, methylation, and one-/two-dimensional NMR spectroscopy indicated that the polysaccharide simultaneously exhibited α/β configurations and a pyranose ring structure, and that it contained 16 types of glycosidic linkages, of which 9 were predominant. Regarding its bioactivity, PFCP-2-1 markedly preserved gastric mucosal integrity; it lowered the ulcer index and improved the gastric epithelial barrier function by increasing serum SOD activity, decreasing MDA levels, and enhancing the expression of PGE2, TGF-α, MUC5AC, as well as tight junction proteins (Occludin and ZO-1) in gastric tissues. Moreover, PFCP-2-1 modulated the gut microbiota by promoting beneficial bacteria and suppressing harmful ones, and, through microbial degradation, generated SCFAs (e.g., acetate and butyrate) that may have indirectly regulated the expression of gastric mucosal defense factors, thus providing a protective effect. However, the acute ethanol model, lack of signaling pathway analysis, and single-dose microbiome data limit the mechanistic depth of this study. Future work should investigate multiple ulcer models, dose-dependent microbiota responses, metabolomics, and specific molecular pathways.

## Figures and Tables

**Table 1:** Analysis of methylated sugar alcohol acetyl ester (PMAAs) of PFCP-2-1.

RT	Methylated Sugar	Mass Fragments (m/z)	Molar Ratio	Type of Linkage
10.541	2,3,4-Me_3_-Rhap	45,58,72,89, 101, 117, 131, 159	0.064	Rhap-(1 →
15.319	2,4-Me_2_-Rhap	43,58,85,89,99, 117, 127, 131, 159,201	0.108	→3)-Rhap-(1 →
17.028	2,3,4,6-Me_4_-Glcp	43,71,87, 101, 117, 129, 145, 161,205	0.064	Glcp-(1 →
17.962	2,3,4,6-Me_4_-Galp	43,71,87, 101, 117, 129, 145, 161,205	0.094	Galp-(1 →
19.192	3-Me_1_-Rhap	43,87, 101, 117, 129, 143, 159, 189	0.042	→2,4)-Rhap-(1→
21.147	2,4,6-Me_3_-Glcp	43,87,99, 101, 117, 129, 161, 173,233	0.105	→3)-Glcp-(1 →
21.501	2,3,6-Me_3_-Galp	43,87,99, 101, 113, 117, 129, 131, 161, 173,233	0.255	→4)-Galp-(1 →
21.897	2,3,6-Me_3_-Glcp	43,87,99, 101, 113, 117, 129, 131, 161, 173,233	0.025	→4)-Glcp-(1 →
22.256	2,4,6-Me_3_-Galp	43,87,99, 101, 117, 129, 161, 173,233	0.021	→3)-Galp-(1 →
23.114	2,3,4-Me_3_-Glcp	43,87,99, 101, 117, 129, 161, 189,233	0.030	→6-Glcp-(1 →
23.823	2,3,4-Me_3_-Galp	43,87,99, 101, 117, 129, 161, 189,233	0.035	→6)-Galp-(1 →
24.474	2,6-Me_2_-Glcp	43,87,97, 117, 159, 185	0.026	→3,4)-Glcp-(1 →
26.804	2,3-Me_2_-Glcp	43,71,85,87,99, 101, 117, 127, 159, 161,201	0.028	→4,6)-Glcp-(1 →
27.208	2,4-Me_2_-Glcp	43,87, 117, 129, 159, 189,233	0.033	→3,6)-Glcp-(1 →
28.692	2,3-Me_2_-Galp	43,71,85,87,99, 101, 117, 127, 159, 161,201,261	0.046	→4,6)-Galp-(1 →
30.673	2,4-Me_2_-Galp	43,87, 117, 129, 159, 189,233	0.024	→3,6)-Galp-(1 →

**Arrows indicate the glycosidic linkages, pointing from the anomeric carbon (the glycosyl donor) to the carbon bearing the hydroxyl group involved in the bond (the glycosyl acceptor).**

**Table 2:** Chemical shift in ^1^H and ^13^C spectrum signals of PFCP-2-1.

	Glycosyl Linkages	H1 C1	H2 C2	H3 C3	H4 C4	H5 C5	H6 C6	CH_3_O
A	→4)-α-D-GalpA-(1 →	5.07 99.08	3.70 68.31	3.95 71.35	4.36 77.86	4.33 71.35	175.03	
B	→4)-α-D-GalpA6Me-(1→	5.02 99.37	3.63 69.40	3.75 71.38	4.08 76.34	4.37 70.48	175.03	3.36 60.79
C	→4,6)-α-D-Galp-(1 →	5.02 99.61	3.63 69.40	3.44 71.56	3.68 78.09	3.85 73.52	3.73 60.92	
D	→4)-α-D-Galp-(1 →	5.10 99.37	4.05 70.04	4.35 77.62	3.71 72.43	3.44 71.56	3.73 60.92	
E	β-D-Galp-(1 →	4.58 103.92	3.53 71.56	4.02 74.17	3.35 71.78	3.90 76.40	3.73 60.92	
F	→3)-β-D-Glcp-(1 →	4.43 103.05	4.02 74.17	3.87 68.74	3.93 68.74	3.78 74.05	3.62 60.70	
G	β-D-Glcp-(1 →	4.63 96.32	3.62 75.04	3.71 75.69	4.01 69.14	3.61 72.43	3.62 60.70	
H	→3)-α-D-Rhap-(1 →	5.18 98.70	4.11 77.64	4.00 72.30	3.50 71.56	3.68 72.60	1.19 16.63	
I	α-D-Rhap-(1 →	5.18 98.70	4.42 78.08	4.20 70.70	3.93 68.74	3.85 68.27	1.24 16.84	

**Arrows indicate the glycosidic linkages, pointing from the anomeric carbon (the glycosyl donor) to the carbon bearing the hydroxyl group involved in the bond (the glycosyl acceptor).**

**Table 3:** SCFA contents (μg/g) of intestinal contents in mice.

	NC	Model	PFCP-2-1L
Acetic acid	20.21 ± 1.56 a	21.25 ± 1.13 a	25.03 ± 0.46 b
Propionic acid	15.51 ± 0.66 a	16.40 ± 0.71 a	16.47 ± 1.65 a
Butyric acid	27.34 ± 0.18 a	25.80 ± 1.18 a	36.14 ± 2.01 b
Isobutyric acid	7.38 ± 0.35 a	7.67 ± 0.094 a	7.83 ± 0.22 a
Valeric acid	31.47 ± 2.48 ab	24.41 ± 4.18 a	37.81 ± 5.15 b

Different lowercase letters represent significant differences (*p* < 0.05).

## Data Availability

The data that support the findings of this study are available upon reasonable request from the corresponding author.

## Abbreviations

The following abbreviations are used in this manuscript:

AFM	Atomic force microscopy
ANOVA	Analysis of variance
CAT	Catalase
COSY	Correlation spectroscopy (^1^H-^1^H correlation spectroscopy)
DEAE-52	Diethylaminoethyl cellulose-52
ELISA	Enzyme-linked immunosorbent assay
F/B	Firmicutes/Bacteroidetes ratio
FDR	False discovery rate
FT-IR	Fourier transform infrared spectroscopy
Gal	Galactose
GalA	Galacturonic acid
GC	Gas chromatography
GC-MS	Gas chromatography–mass spectrometry
Glc	Glucose
GPC	Gel permeation chromatography
H&E	Hematoxylin and eosin
HPSEC	High-performance size-exclusion chromatography
HSQC	Heteronuclear single quantum coherence
IC	Ion chromatography
IOD	Integrated optical density
KM	Kunming (mice)
MDA	Malondialdehyde
MUC5AC	Mucin 5AC
MW	Molecular weight
NC	Normal control
NMR	Nuclear magnetic resonance
NOESY	Nuclear Overhauser effect spectroscopy
OMe	Omeprazole
PBS	Phosphate-buffered saline
PCA	Principal component analysis
PCoA	Principal coordinate analysis
PFCP	Polysaccharide of Finger Citron-pickled products
PFCP-2-1	Polysaccharide-2-1 of Finger Citron-pickled products
PGE2	Prostaglandin E2
PMAAs	Partially methylated alditol acetates
Rha	Rhamnose
SCFAs	Short-chain fatty acids
SD	Standard deviation
SEM	Scanning electron microscopy
SOD	Superoxide dismutase
SPF	Specific pathogen-free
TGF-α	Transforming growth factor-alpha
TFA	Trifluoroacetic acid
ZO-1	Zonula occludens-1
